# Sexual dimorphism in relation to adipose tissue and intrahepatocellular lipid deposition in early infancy

**DOI:** 10.1038/ijo.2015.4

**Published:** 2015-02-17

**Authors:** C Gale, K M Logan, S Jeffries, J R C Parkinson, S Santhakumaran, S Uthaya, G Durighel, A Alavi, E L Thomas, J D Bell, N Modi

**Affiliations:** 1 Section of Neonatal Medicine, Chelsea and Westminster Hospital Campus, Imperial College London, London, UK; 2 Robert Steiner MRI Unit, Hammersmith Campus, Imperial College London, London, UK; 3 Radiology Department, St Mary's Hospital, London, UK; 4 Department of Life Sciences, University of Westminster, London, UK

## Abstract

Sexual dimorphism in adiposity is well described in adults, but the age at which differences first manifest is uncertain. Using a prospective cohort, we describe longitudinal changes in directly measured adiposity and intrahepatocellular lipid (IHCL) in relation to sex in healthy term infants. At median ages of 13 and 63 days, infants underwent quantification of adipose tissue depots by whole-body magnetic resonance imaging and measurement of IHCL by *in vivo* proton magnetic resonance spectroscopy. Longitudinal data were obtained from 70 infants (40 boys and 30 girls). In the neonatal period girls are more adipose in relation to body size than boys. At follow-up (median age 63 days), girls remained significantly more adipose. The greater relative adiposity that characterises girls is explained by more subcutaneous adipose tissue and this becomes increasingly apparent by follow-up. No significant sex differences were seen in IHCL. Sex-specific differences in infant adipose tissue distribution are in keeping with those described in later life, and suggest that sexual dimorphism in adiposity is established in early infancy.

## Introduction

Sexual dimorphism in adipose tissue distribution and intrahepatocellular lipid (IHCL) are well described in adults.^1, 2^ Adipose tissue distribution, specifically the accumulation of internal abdominal (IA) (or visceral) adipose tissue is strongly associated with cardiovascular and metabolic disease in adults^3^ (especially adult men^4^) and children.^5^ The age at which sex differences in the distribution of adiposity begin to manifest remains unclear. Sex differences are established in adolescent,^6^ pre-adolescent^7^ and pre-school children,^8^ implicating early childhood or infancy as the period of divergence, although there is a paucity of data describing adipose tissue distribution early in life. Here, we describe the longitudinal changes in directly measured adiposity and hepatic lipid that occur between birth and 2–3 months in healthy term babies in relation to infant sex.

## Methods

The results of this study represent secondary analyses of two previously described cohorts (cohort one recruited in November 1999–October 2001 at the Hammersmith Hospital; cohort two recruited in March 2010–May 2012 at Chelsea and Westminster Hospital, both in London, UK) recruited to examine the association between method of feeding and infant adiposity.^9^ Participants were healthy, full-term, appropriate weight for gestational age infants that underwent longitudinal measurements at two time points, baseline (shortly after birth) and follow-up (6–12 weeks). Infants of diabetic mothers and smokers were excluded. Maternal pre-pregnancy body mass index (BMI) was determined from pre-pregnancy weight obtained by maternal recall and maternal height measured at pregnancy booking. Data from both cohorts are presented together. The study was approved by the National Research Ethics Committee (10/H0713/5).

Anthropometric measures and magnetic resonance investigations were performed with infants in natural sleep, as previously described.^9, 10^ Whole-body magnetic resonance images (MRIs) were acquired on a Phillips (Amsterdam, Netherlands) 1.5 Tesla system using a T_1_-weighted rapid-spin-echo sequence (repetition time of 500 ms, echo time of 17 ms, echo train length of 3) using a Q body coil. The slice thickness was 5 mm and interslice difference was 5 mm. Voxel size was 0.31 × 0.31 × 0.31 cm. Scanning time was ~15 min. Analysis of all magnetic resonance images was undertaken independently of the investigators and blind to participant identity and sex by VardisGroup, (London, UK, www.vardisgroup.com), using an image segmentation program (SliceOmatic, Tomovision, Montreal, Canada). Total adipose tissue volume was calculated as the sum of six individually quantified adipose tissue compartments (IA, internal non-abdominal, deep subcutaneous abdominal, deep subcutaneous non-abdominal, superficial subcutaneous abdominal, superficial subcutaneous non-abdominal) as previously described,^9^ Supplementary Figure 1. We calculated the ratio IA to subcutaneous abdominal, where SCA comprised the sum of the superficial subcutaneous abdominal and deep subcutaneous abdominal compartments. In adults, the ratio IA/SCA correlates more strongly with cardiometabolic risk than the IA compartment alone.^11^

IHCL content was quantified as previously described.^9^ Briefly, proton magnetic resonance spectra were acquired at 1.5T from the right lobe of the liver using a point-resolved spectroscopy sequence, repetition time 1500 ms, echo time 135 ms, without water saturation and with 128 signal averages. Spectra were analyzed in the time domain using the AMARES algorithm included in the MRUI software package (http://sermn02.uab.es/mrui/mrui_download/) by a single investigator (ELT) blinded to infant identity and sex. Peak areas for all resonances were obtained and lipid resonances quantified with reference to water resonance, after correcting for *T*_1_ and *T*_2_. Hepatic water, known to be relatively constant,^12^ was used as an internal standard. The results are presented as the ratio IHCL CH_2_/water.

### Statistical analyses

Analyses were performed using SPSS version 20 (IBM Corporation, Armonk, NY, USA). Statistical significance was defined as *P*<0.05. Where data were not normally distributed (*P*<0.05 from the Shapiro–Wilk test), a natural log transformation was undertaken. Where data remained non-normal in distribution, non-parametric tests were used.

We compared girls and boys using multivariable regression to examine total adipose tissue, each adipose tissue depot and IHCL, with infant weight as a covariate to adjust for body size. The ratio IA/SCA is a measure of metabolic load; to compare boys and girls, the optimal index for infancy^13^ (IA/SCA^0.6^) was calculated. This index has not to our knowledge been correlated with metabolic variables in infancy. This index is correlated with body weight at follow-up (*P*<0.05; Spearman's correlation), therefore multivariable regression was used with infant weight as a covariate. As ethnicity and maternal BMI are associated with adiposity in infancy,^14, 15^ sensitivity analyses were undertaken after exclusion of non-Caucasian infants and with adjustment for maternal BMI. No significant association has been detected between method of infant feeding and directly measured adipose tissue,^9^ therefore the feeding group was not included in the multivariable adjustment.

## Results

Longitudinal adiposity data were acquired from 70 infants (40 boys and 30 girls); these data have been previously described.^9^ Mean (SD) gestation, birth weight and birth weight SDS scores were 39.8 (1.4) weeks, 3.441 (0.397) kg and −0.12 (0.79) for boys and 40.0 (1.2) weeks, 3.350 (0.404) kg and −0.02 (0.85) for girls. Mean (s.d.) maternal pre-pregnancy maternal BMI was 22.8 (2.4) kg m^−2^ for boys and 24.3 (5.3) kg m^−2^ for girls. Demographic data and unadjusted adiposity data are presented by infant sex in Table 1.

Adjusted analyses are shown in Table 2. After adjusting for body size, girls had significantly more total and superficial subcutaneous abdominal adipose tissue than boys in early infancy (median age 13 days). At follow-up (median age 63 days), all adjusted adipose depots were larger in girls; these differences reached statistical significance for internal non-abdominal, deep subcutaneous abdominal, superficial subcutaneous abdominal and superficial subcutaneous non-abdominal adipose tissue depots. Sensitivity analyses with adjustment for maternal BMI and after exclusion of non-Caucasian infants did not alter these findings (Supplementary Table 1). No significant difference was detected in the ratio IA:SCA^0.6^ between boys and girls (*P*=0.38 at baseline; *P*=0.43 at follow-up). No sex-specific differences in IHCL were detectable at baseline or follow-up.

## Discussion

We confirm, in this longitudinal cohort of healthy term babies, that sex-specific differences in total adiposity are present from shortly after birth. These differences become more pronounced by 2–3 months by which time a difference in adipose tissue distribution, characterised by higher subcutaneous adipose tissue volumes in girls, is detectable. The greater relative adiposity in girls compared with boys is in keeping with recognised sex differences,^16, 17^ but to our best knowledge a sex difference in adipose tissue distribution in early infancy has not been described previously in longitudinal measurements. The strengths of this study include the use of an established gold standard method, whole-body MRI,^18^ to quantify adipose tissue distribution in combination with blinded image analysis. Important limitations include the secondary nature of this study and that initial measurements were taken at a median of 13 days, so we are unable to comment on differences present at birth.

Sex-specific differences in adult adipose tissue distribution were first recognised by Vague *et al.*;^1^ these differences, namely more IA adipose tissue and less subcutaneous adipose tissue in men, are implicated in the higher cardiovascular risk seen in men compared with pre-menopausal women.^4^ There are limited data on the ontogeny of sex-specific differences in adipose tissue distribution. We have previously noted small differences in newborns with greater total adipose tissue, and larger abdominal and non-abdominal compartments in girls.^15^ By the age of 5 years, boys have more IA adipose tissue than girls but a similar amount of SCA adipose tissue,^8^ and by puberty, differences in keeping with those described in adults are established.^7^ Our observations suggest that sexual divergence in adipose tissue distribution occurs in early infancy. Adipose tissue is considered an important energy reserve in the weaning period,^19^ and the greater relative adiposity of baby girls may contribute to the survival advantage they manifest in early life.^20^ Sex-specific differences in IHCL are described in adults,^2^ but we find no evidence for such a difference in infancy. We have previously commented on the substantial increase in IHCL during early infancy suggesting that the high content is not of pathological relevance as in older age groups.^8^

Sex-related adiposity differences have been attributed to sex hormones; testosterone mediates deposition of adipose tissue in abdominal regions and oestrogen in gluteo-femoral regions.^21^ Androgen receptors are more densely expressed in visceral than in subcutaneous adipose tissue.^22^ There is surge shortly after birth in luteinising hormone (LH), follicle-stimulating hormone (FSH) and sex hormones, although levels are generally low in childhood. This has been termed a ‘minipuberty of early infancy'^23^ in boys FSH, LH and testosterone concentrations increase during the first week before decreasing by about 6 months, whereas in girls FSH, LH and oestrogen increase shortly after birth and remain raised until 2–3 years.^23^ The gender-related divergence in adipose tissue distribution we observed may be mediated by these hormonal fluctuations.

Adipose tissue quantity and distribution are important determinants of health. Data presented here indicate that the sex-specific divergence seen in adults develops during early infancy. If confirmed this supports the importance of the infant period in mediating trajectories of adipose tissue development.

## Figures and Tables

**Table 1:** Anthropometric data, adipose tissue compartment volumes and IHCL at baseline and follow-up by infant sex

	*Baseline*		*Follow-up*	
	*Boys*	*Girls*	*Boys*	*Girls*
*n*	40	30	40	30
Age (days)	13 [7–20]	13 [5–18]	64 [54–70]	62 [56–71]
Weight (kg)	3.637 (0.489)	3.546 (0.518)	5.492 (0.687)	5.219 (0.533)
Weight SDS^a^	−0.25 (0.94)	−0.17 (0.78)	−0.12 (0.99)	0.13 (0.55)
*n*	40	29	39	30
Total AT (l)	0.709 [0.608–0.932]	0.730 [0.626–0.906]	1.548 [1.278–1.801]	1.485 [1.345–1.817]
Superficial subcutaneous abdominal AT (l)	0.098 [0.075–0.122]	0.104 [0.087–0.0139]	0.257 [0.198–0.316]	0.258 [0.223–0.326]
Superficial subcutaneous non-abdominal AT (l)	0.512 [0.436–0.692]	0.515 [0.461–0.653]	1.124 [0.935–1.270]	1.085 [0.927–1.263]
Deep subcutaneous abdominal AT	0.015 [0.010–0.021]	0.017 [0.011–0.022]	0.037 [0.028–0.046]	0.044 [0.031–0.053]
Deep subcutaneous non-abdominal AT (l)	0.012 [0.010–0.022]	0.013 [0.010–0.016]	0.020 [0.016–0.023]	0.020 [0.015–0.024]
Internal abdominal AT (l)	0.017 [0.013–0.022]	0.017 [0.012–0.027]	0.030 [0.022–0.038]	0.030 [0.023–0.042]
Total internal AT (l)	0.071 [0.055–0.091]	0.069 [0.060–0.097]	0.118 [0.096–0.148]	0.130 [0.110–0.156]
IA/SCA^a^	0.16 [0.12–0.20]	0.14 [0.11–0.16]	0.10 [0.08–0.15]	0.10 [0.08–0.13]
IA/SCA^0.6^	0.067 (0.021)	0.062 (0.022)	0.069 (0.024)	0.067 (0.025)
*n*	31	23	31	23
IHCL^b^	0.656 [0.425–1.965]	1.142 [0.667–1.644]	1.818 [1.397–2.168]	2.123 [1.278–3.229]

Abbreviations: AT, adipose tissue; IA, internal abdominal; IHCL, intrahepatocellular lipid; SCA, subcutaneous abdominal.

Values are median and [interquartile range] or mean (s.d.) and units are as specified except

a that has no units and

b that is ratio CH_2_/water.

**Table 2:** Mean difference in adiposity (l) and IHCL (ratio CH_2_:H_2_O) at follow-up, boys compared with girls (adjusted for weight at imaging)

	*Baseline*			*Follow-up*		
	R^*2*^	*Adjusted mean difference*	P	R^*2*^	*Adjusted mean difference*	P
*n*		69			69	
Total AT	0.58	−0.075 (−0.154, 0.001)	0.05	0.63	−0.193 (−0.312, −0.074)	0.002
Superficial subcutaneous abdominal AT	0.48	−0.018 (−0.033, −0.002)	0.02	0.44	−0.039 (−0.070, −0.007)	0.02
Superficial subcutaneous non-abdominal AT	0.59	−0.046 (−0.100, 0.008)	0.09	0.64	−0.124 (−0.205, −0.043)	0.003
Deep subcutaneous abdominal AT	0.43	−0.002 (−0.004, 0.001)	0.23	0.25	−0.007 (−0.014, −0.001)	0.04
Deep subcutaneous non-abdominal AT	0.23	−0.002 (−0.003, 0.000)	0.10	*0.22	−9.6%*(−26.1%, 10.6%)	0.32**
Internal abdominal AT	0.10	−0.001 (0.004, 0.003)	0.77	0.01	−0.001 (−0.009, 0.006)	0.69
Internal non-abdominal AT	0.33	−0.007 (−0.015, 0.001)	0.07	0.40	−0.019 (−0.032, −0.005)	0.007
Ratio (IA/SCA)^0.6^	0.02	0.01 (−0.01, 0.02)	0.37	0.07	0.01 (−0.01, 0.02)	0.38
*n*		54			54	
IHCL	0.02*	−6.4% (−39.7%, 45.2%)*	0.76**	0.04*	−31.3% (−60.7%, 20.1%)*	0.18**

Abbreviations: AT, adipose tissue; IA, internal abdominal; IHCL, intrahepatocellular lipid; SCA, subcutaneous abdominal.

*P* represents significance of infant sex in overall analyses. Data are mean and (95% confidence intervals) and *P*-values from multivariable regression, except where * denotes percentage difference (95% confidence intervals) following log transformation and ** denotes *P*-values from multivariable regression of natural log-transformed data (performed using transformed data due to non-normal distribution of residuals).

## Notes

CG received support from Pfizer Nutrition to attend an educational conference. In the last 5 years NM has received consultancy fees from Ferring Pharmaceuticals, speaker honorarium for an educational meeting funded by Nestle International in which they had no organisational involvement and grants from the Medical Research Council, National Institute of Heath Research, Westminster Children's Trust Fund, Child Growth Foundation, HCA International, Bliss, British Heart Foundation, and Department of Health. The remaining authors declare no conflict of interest.
