# Supplementary material for: Sexual dimorphism in relation to adipose tissue and intrahepatocellular lipid deposition in early infancy
Source: Int J Obes (Lond). 2015 Feb 17;39(4):629–32. doi: 10.1038/ijo.2015.4 (PMC4389723; doi:10.1038/ijo.2015.4)
Supplement: Supplementary Table 1 [file ijo20154x1.doc]

**Total Adipose Tissue**

Superficial Subcutaneous

Adipose Tissue

Deep Subcutaneous

Adipose Tissue

Internal

Adipose Tissue

Superficial Subcutaneous

Abdominal

Superficial Subcutaneous Non-Abdominal

Deep Subcutaneous Abdominal

Deep Subcutaneous Non-Abdominal

Internal Abdominal

**(IA)**

Internal

Non-Abdominal

Subcutaneous

Abdominal

**(SCA)**
