# Supplementary material for: Sexual dimorphism in relation to adipose tissue and intrahepatocellular lipid deposition in early infancy
Source: Int J Obes (Lond). 2015 Feb 17;39(4):629–32. doi: 10.1038/ijo.2015.4 (PMC4389723; doi:10.1038/ijo.2015.4)
Supplement: Supplementary Table 2 [file ijo20154x2.doc]

**Supplemental table 1**

|  | **Baseline** | | | **Follow-up** | | |
| --- | --- | --- | --- | --- | --- | --- |
|  | **R^2^** | **Mean difference** | ***p*** | **R^2^** | **Mean difference** | ***p*** |
| *n* |  | 38 |  |  | 39 |  |
| Total AT | 0.76 | -0.142 (-0.215, -0.068) | <0.001 | 0.68 | -0.318 (-0.483, 0.153) | <0.001 |
| Superficial Subcutaneous Abdominal AT | 0.61 | -0.028 (-0.045, -0.011) | 0.002 | 0.57 | -0.086 (-0.127, -0.044) | <0.001 |
| Superficial Subcutaneous Non-Abdominal AT | 0.77 | -0.100 (-0.151, -0.050) | <0.001 | 0.69 | -0.203 (-0.314, -0.091) | 0.001 |
| Deep Subcutaneous Abdominal AT | 0.44 | -0.002 (-0.006, 0.002) | 0.23 | 0.27 | -0.009 (-0.017, 0.000) | 0.05 |
| Deep Subcutaneous Non-abdominal AT | 0.27 | -0.002 (-0.004, 0.00) | 0.11 | 0.42* | -7.7% (-27.8%, 17.9%)* | 0.51** |
| Internal Abdominal AT | 0.14 | -0.001 (-0.005, 0.004) | 0.82 | 0.15 | -0.004 (-0.014, 0.007) | 0.49 |
| Internal Non-Abdominal AT | 0.47 | -0.008 (-0.018, 0.003) | 0.14 | 0.30 | -0.018 (-0.038, 0.003) | 0.09 |
| Ratio (IA/SCA)^0.6^ | 0.04 | 0.01 (-0.01, 0.02) | 0.04 | 0.07 | 0.01 (-0.01, 0.02) | 0.41 |
| *n* |  | 36 |  |  | 39 |  |
| IHCL | 0.04* | -32.3% (-68.8%, 47.3%)* | 0.31** | 0.05* | -20.3% (-52.7%, 34.2%)* | 0.38** |

**Supplemental table 1: Mean difference in adiposity (litres) and IHCL (ratio CH_2_:H_2_O) at follow-up, girls compared to boys, non-Caucasian babies excluded and adjusted for weight at imaging and maternal BMI.** *p* represents significance of infant sex in overall analyses. Data are mean and (95% confidence intervals) and *p* values from multivariable regression, except where * percentage difference (95% confidence intervals) following log transformation and ** denotes *p* values from multivariable regression of natural log transformed data (performed using transformed data due to non-normal distribution of residuals). n: number; AT: Adipose Tissue; IA: Internal Abdominal; SCA: Subcutaneous Abdominal
